# Peimine Alleviates DSS-Induced Colitis by Modulating Gut Microbiota and Attenuating Inflammation and Oxidative Stress

**DOI:** 10.3390/ijms262211203

**Published:** 2025-11-20

**Authors:** Xuke Guan, Deping Han, Haojie Sha, Moyue Yao, Jiaying Zhang, Guangyao Zhang, Yibing Wu, Dingding Su, Qing Yang

**Affiliations:** 1 Shandong Laboratory of Advanced Agricultural Sciences in Weifang, Peking University Institute of Advanced Agricultural Sciences, Weifang 261325, China; 2 College of Veterinary Medicine, Hunan Agricultural University, Changsha 410128, China

**Keywords:** peimine, aged garlic, ulcerative colitis, gut microbiota, mucosal immunology

## Abstract

Peimine (PM), a steroidal alkaloid derived from aged garlic (*Allium sativum* L.), demonstrates potent therapeutic efficacy against ulcerative colitis (UC) through multi-target mechanisms. Integrating network pharmacology and in vivo validation, we reveal that PM suppresses colitis by concurrently inhibiting PI3K-AKT, JAK-STAT, and HIF-1 signaling pathways—key drivers of inflammation and oxidative stress. In a murine model of dextran sulfate sodium (DSS)-induced UC, oral PM administration (4 mg/kg) significantly attenuated disease severity, evidenced by reduced disease activity index, restored colon length, and improved epithelial barrier integrity. PM treatment diminished pro-inflammatory cytokines TNF-α (4.2-fold) and IL-6 (3.1-fold) and oxidative damage while reshaping gut microbiota composition to enrich beneficial taxa (*Akkermansia muciniphila*, *Lactobacillus* spp.). Critically, PM rescued fecal short-chain fatty acid (SCFA) production (acetate, propionate, butyrate), directly linking microbial remodeling to mucosal healing. These findings establish PM as a novel natural compound targeting inflammation-redox-microbiota crosstalk, offering a promising pharmacological strategy for UC management.

## 1. Introduction

Inflammatory bowel disease (IBD), encompassing Crohn’s disease (CD) and ulcerative colitis (UC), is characterized by chronic intestinal inflammation [1]. While the incidence of IBD has stabilized in many Western nations, it exhibits a concerning upward trajectory in China [1,2]. For instance, the prevalence of UC in China surged from 11.6 per 100,000 people annually between 1990 and 2003 to 19.04 per 100,000 in Eastern China by 2016, with a particularly marked increase observed among younger populations [3]. Concurrently, IBD imposes a significant healthcare burden in the USA and Europe, affecting over 1 million and 2.5 million individuals, respectively, and Projections indicate that the number of IBD cases in China could reach 1.5 million by 2025 [4].

The pathogenesis of UC is multifactorial, arising from complex interactions between genetic susceptibility, immune dysregulation, epithelial barrier dysfunction, gut microbiota dysbiosis, and environmental factors [5,6]. This dysregulation culminates in aberrant immune activation and compromised intestinal barrier integrity, driving chronic inflammation and tissue damage. Current therapeutic strategies primarily aim to manage symptoms and induce remission [7,8], utilizing agents such as aminosalicylates (e.g., 5-aminosalicylic acid (5-ASA)) [9], glucocorticoids (GCs) for moderate-severe flares (though limited by significant adverse effects and unsuitability for long-term use) [10], immunosuppressants (e.g., thiopurines like 6-mercaptopurine) [11], and biologics or small molecules (e.g., JAK inhibitors, anti-TNF-α, anti-integrin antibodies) [12,13] targeting specific inflammatory pathways. Despite these options, approximately 10% of patients ultimately require colectomy [14], particularly pediatric cases where early intervention is often recommended [15]. Critically, remission and cure rates remain suboptimal, estimated at only 20–30% [14]. This significant unmet clinical need underscores the urgency of developing novel therapeutic approaches that directly target the core pathogenic mechanisms of UC, including persistent inflammation, oxidative stress, and gut microbiota imbalance.

Aged garlic—a fermented derivative of raw garlic—is well-documented for its potent antioxidant, antimicrobial, and anti-inflammatory properties [16]. Chemical analysis in our study identified peimine (PM) as a key constituent significantly upregulated in aged garlic relative to fresh garlic. Network pharmacology further implicated PM as a central mediator of aged garlic’s therapeutic effects against colitis, highlighting its potential as a critical bioactive component for IBD management. However, while PM has demonstrated anti-inflammatory efficacy in diverse pathological contexts, including acute lung injury [17], osteoarthritis [18], chronic obstructive pulmonary disease, and cancer [19], its specific role and mechanisms in colitis remain largely unexplored. To address this gap, we employed a murine UC model to systematically investigate PM’s mechanisms in alleviating colonic inflammation and damage. This study provides the first comprehensive analysis of PM’s multi-faceted mechanisms in colitis, integrating network pharmacology with in vivo validation to elucidate its therapeutic potential for UC.

## 2. Results

### 2.1. Network Pharmacology and Molecular Docking Identify Key Targets and Pathways for Peimine in UC

**Figure 1:**
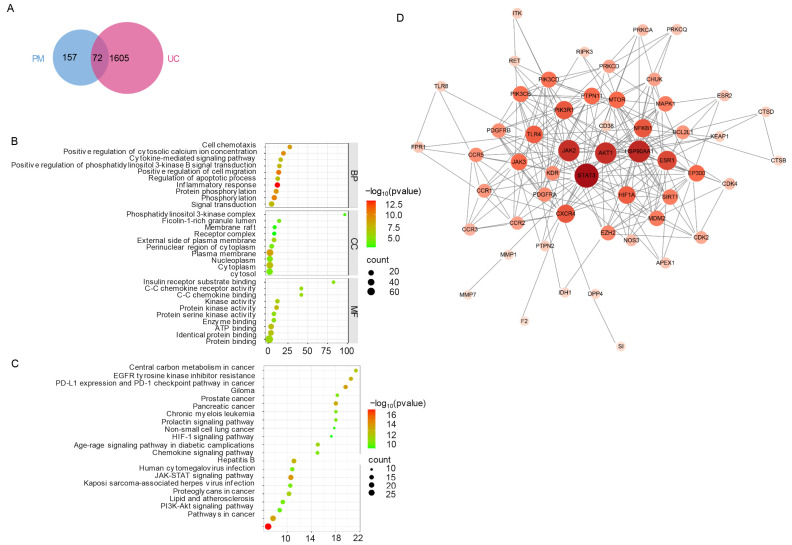
Network construction and analysis of PM for alleviating UC. (**A**) Venn diagram of PM and UC targets. (**B**) GO and (**C**) KEGG enrichment analysis, and (**D**) PPI (Protein-Protein interaction) network of common targets. The shade of color represents the number of interacting protein molecules. PM: peimine. UC: ulcerative colitis.

Network pharmacology analysis identified 229 potential peimine (PM) targets from the SwissTargetPrediction database and 1677 ulcerative colitis (UC)-related targets from the GeneCards database. Intersection analysis revealed 72 common targets shared between PM and UC (Figure 1A), indicating potential key mediators of PM’s effects against UC. Gene Ontology (GO) enrichment analysis using DAVID identified 332 significantly enriched terms (*p* < 0.05), comprising 221 biological processes (BPs), 41 cellular components (CCs), and 60 molecular functions (MFs). The top enriched BP terms included inflammatory response, positive regulation of cell migration, and phosphorylation, suggesting PM’s potential role in mitigating inflammation and modulating cell signaling relevant to UC (Figure 1B). Enriched CC terms, such as plasma membrane, cytoplasm, and external side of plasma membrane, pointed to the significance of membrane-associated and cytoplasmic processes in PM’s action (Figure 1B). MF analysis highlighted enrichment in kinase activity, ATP binding, and chemokine receptor binding, further implicating PM in immune regulation and signal transduction (Figure 1B). Kyoto Encyclopedia of Genes and Genomes (KEGG) pathway enrichment analysis identified several significantly enriched pathways linked to immune regulation, cellular signaling, and metabolic adaptation (Figure 1C).

**Figure 2:**
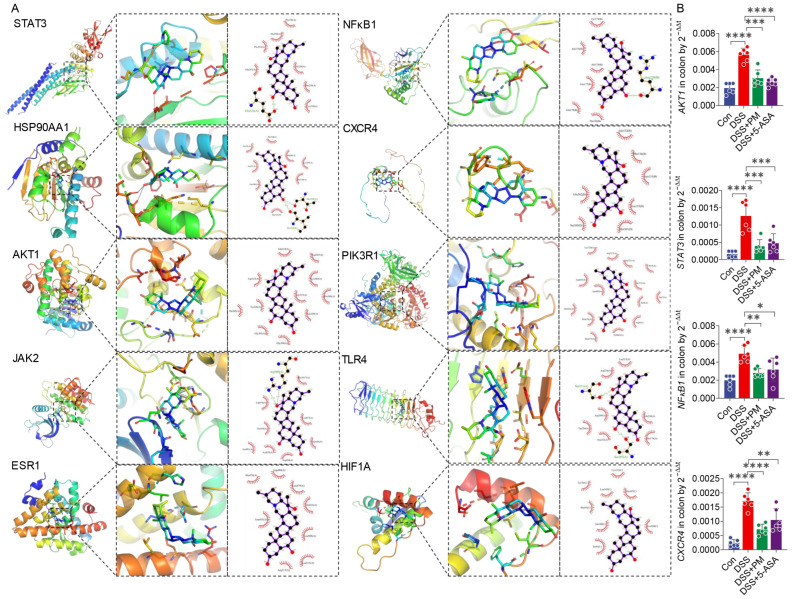
Docking simulation results of peimine (PM) and key targets. (**A**) STAT3, HSP90AA1, AKT1, JAK2, ESR1, NFκB1, CXCR4, PIK3R1, TLR4, and HIF1A. (**B**) Gene expressions of *AKT1*, *STAT3*, *NFκB1*, and *CXCR4* in the colon by qPCR. * *p* ≤ 0.05, ** *p* ≤ 0.01, *** *p* ≤ 0.001, and **** *p* ≤ 0.0001 indicate significant differences between different groups.

Key pathways included the PI3K-Akt signaling pathway, Chemokine signaling pathway, and JAK-STAT signaling pathway, strongly supporting PM’s anti-inflammatory and immunomodulatory potential. Additionally, enrichment of the HIF-1 signaling pathway and the AGE-RAGE signaling pathway in diabetic complications suggested PM might also influence metabolic responses and adaptation to hypoxic stress, processes pertinent to UC pathology. Collectively, these analyses indicate that PM exerts its potential therapeutic effects in UC through modulation of multiple interconnected pathways governing inflammation, immune response, and cellular homeostasis. To elucidate the core interactions, a protein-protein interaction (PPI) network was constructed using the STRING database for the 72 common targets (Figure 1D). Molecular docking analysis of key hub targets identified by network centrality (Figure 2, Table 1) revealed strong binding affinities between PM and STAT3 (highest degree = 28, closeness centrality = 0.68; binding energy = −8.1 kcal/mol), AKT1 (binding energy = −9.4 kcal/mol), and PIK3R1 (binding energy = −9.6 kcal/mol). The high docking scores for these targets, which are central players in the PI3K-Akt and JAK-STAT pathways, provide computational validation that PM likely modulates these critical signaling hubs to regulate inflammatory responses, cell survival, and immune function in UC.

### 2.2. PM Attenuates DSS-Induced Colitis via Suppressing Inflammation and Restoring Intestinal Barrier Integrity

**Figure 3:**
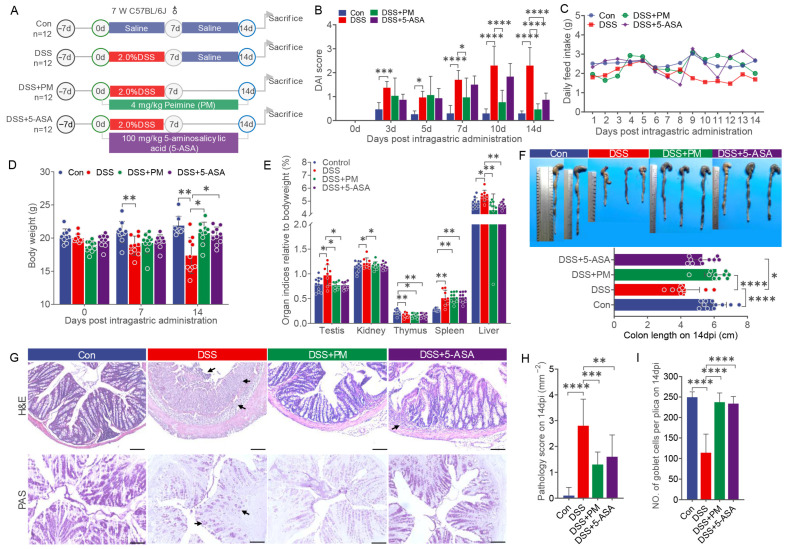
Oral PM alleviated DSS-induced experimental colitis. (**A**) Diagram illustrating the mouse model of colitis employed in this study. (**B**) DAI score, DAI: disease activity index; (**C**) daily food intake, (**D**) body weight, (**E**) different organ indices, and (**F**) length of colon from each group after treatment. (**G**) H&E- and PAS-stained colon sections from different groups. Arrows indicate the pathological changes and goblet cells. Scale bar = 100 μm. (**H**) Pathological scores of colon sections from different groups. (**I**) Number of goblet cells from PAS stain in each group. * *p* ≤ 0.05, ** *p* ≤ 0.01, *** *p* ≤ 0.001, and **** *p* ≤ 0.0001 indicate significant differences between different groups. PM: peimine; H&E: hematoxylin & eosin; PAS: Periodic acid-Schiff.

To evaluate the protective effects of peimine (PM) against ulcerative colitis, we established a DSS-induced colitis model in mice. Animals received 2.0% DSS in drinking water for 7 days, followed by 14 days of daily oral administration of PM (4 mg/kg) or 5-aminosalicylic acid (5-ASA, 100 mg/kg) (Figure 3A). Compared to DSS-treated controls, PM administration significantly attenuated colitis severity, as demonstrated by a marked reduction in the disease activity index (DAI)—a composite measure of weight loss, stool consistency, and rectal bleeding (Figure 3B). Concurrently, PM improved clinical parameters including feed intake and body weight recovery (Figure 3C,D), and attenuated DSS-induced colon shortening (Figure 3F). Systemic pathophysiological alterations, reflected by increased organ indices of the thymus, spleen, testes, kidneys, and liver in DSS mice, were significantly ameliorated by both PM and 5-ASA treatments (Figure 3E).

Histological assessment further revealed that PM and 5-ASA treatment markedly mitigated colonic damage. Both agents reduced inflammatory cell infiltration across the lamina propria, submucosa, and muscularis layers while preserving epithelial integrity and restoring goblet cell populations (Figure 3G–I).

**Figure 4:**
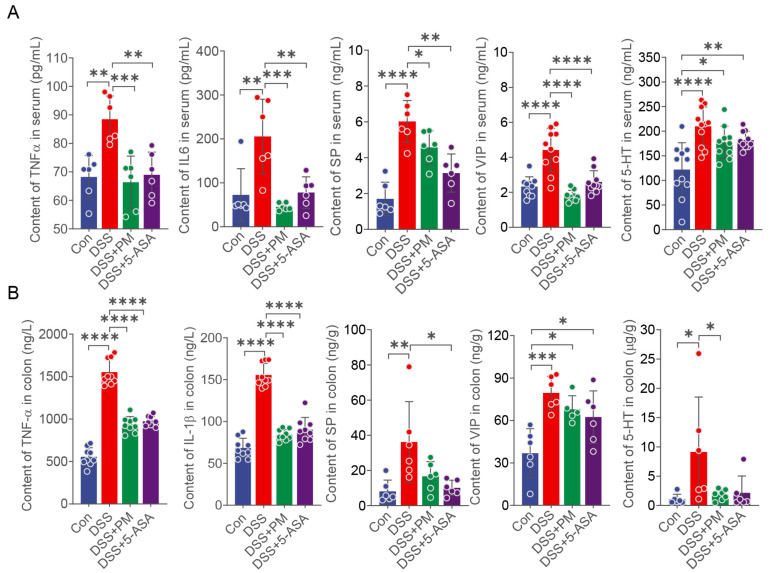
Oral PM attenuated DSS-induced inflammation. TNF-α, IL6, SP, VIP and 5-HT in the (**A**) serum and (**B**) colon. * *p* ≤ 0.05, ** *p* ≤ 0.01, *** *p* ≤ 0.001, and **** *p* ≤ 0.0001 indicate significant differences between different groups.

We next quantified systemic and intestinal inflammatory responses. PM effectively suppressed DSS-induced elevations in serum pro-inflammatory cytokines (TNF-α, IL-6) and neurogenic factors (substance P [SP], vasoactive intestinal peptide [VIP], 5-hydroxytryptamine [5-HT]) (Figure 4A). Similarly, in colonic tissues, PM significantly reduced concentrations of TNF-α, IL-1β, SP, VIP, and 5-HT (Figure 4B). No significant alterations in other cytokines were observed across groups in serum or colon (Figure S2).

Collectively, these results demonstrate that oral PM administration alleviates DSS-induced colitis by suppressing mucosal inflammation, restoring intestinal barrier integrity, and modulating systemic inflammatory responses.

### 2.3. PM Attenuates Oxidative Stress and Restores Mucosal Barrier Integrity

**Figure 5:**
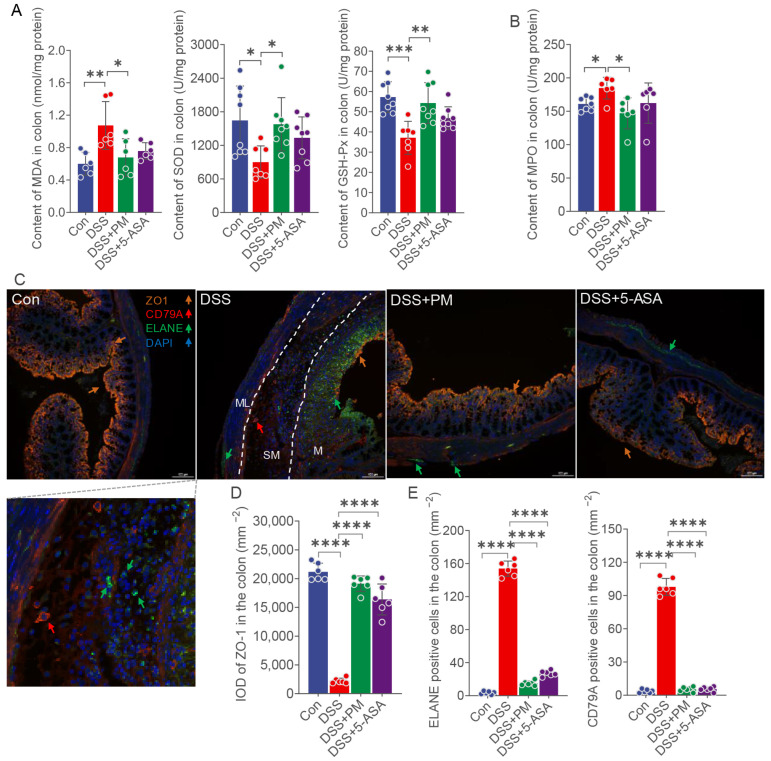
Oral PM attenuated oxidative stress and enhanced mucosa integrity. (**A**) Concentrations of SOD, GSH-Px, and MDA in the colon. (**B**) Content of MPO in the colon. (**C**) Expressions of ZO-1 (orange), CD79A (red), ELANE (green), and DAPI (blue) in the colon. Scale bar = 100 μm. (**D**) IOD of ZO-1 in each group. (**E**) The numbers of CD79A- and ELANE-positive cells in each group. * *p* ≤ 0.05, ** *p* ≤ 0.01, *** *p* ≤ 0.001, and **** *p* ≤ 0.0001 indicate significant differences between different groups. IOD: integral optical density.

To evaluate PM’s impact on oxidative stress, we measured key biomarkers—total superoxide dismutase (T-SOD), glutathione peroxidase (GSH-Px), myeloperoxidase (MPO), and malondialdehyde (MDA)—in colonic tissues. DSS challenge significantly suppressed colonic T-SOD and GSH-Px activity while elevating MDA levels compared to controls; these perturbations were substantially reversed by both PM and 5-ASA treatment (Figure 5A). Similarly, DSS-induced MPO upregulation was markedly attenuated by PM (Figure 5B).

We next assessed mucosal barrier integrity via tight junction protein ZO-1 expression. DSS administration triggered severe epithelial damage, including necrosis, exfoliation, and mucosal thinning in ulcerated regions, accompanied by a dramatic loss of ZO-1 expression across the epithelium and muscular layers (Figure 5C,D). Concurrently, robust infiltration of neutrophils (ELANE+) and B cells (CD79A+) permeated the mucosa (M), submucosa (SM), and muscular layer (ML) (Figure 5D). Oral PM and 5-ASA significantly mitigated these pathological changes: restoring epithelial architecture, enhancing ZO-1 expression, and reducing immune cell infiltration (Figure 5C,D).

Collectively, these data demonstrate that PM alleviates DSS-induced colitis by counteracting oxidative stress and preserving mucosal barrier integrity.

### 2.4. PM Restores Gut Microbiota Homeostasis and Metabolic Function in Colitis

**Figure 6:**
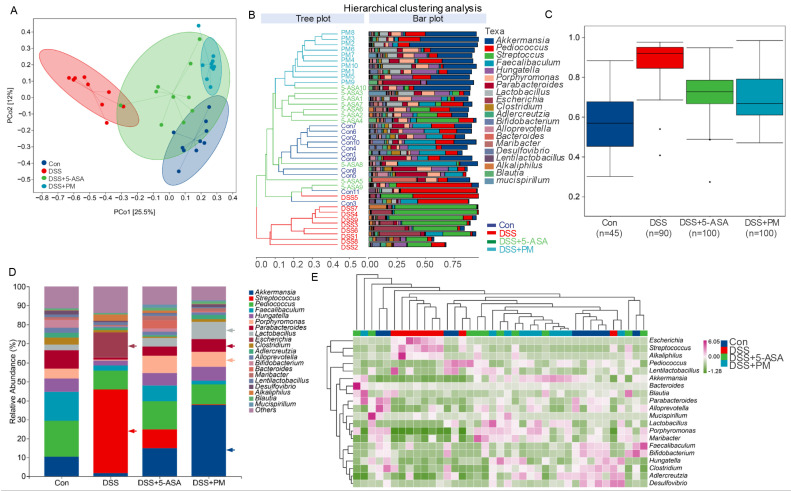
The relative abundance of fecal bacteria among different groups. (**A**) Beta diversity of fecal bacteria by principal coordinate analysis. (**B**) Clustering analysis of top 20 fecal bacterial genera. (**C**) The relative abundance of differential bacterial species in each group. (**D**) The relative abundance of the top 20 bacterial genera in each group. (**E**) The Heatmap showed the composition and relative abundance of the top 20 fecal bacteria among groups.

To determine whether peimine (PM) modulates gut microbiota dysbiosis in colitis, we performed 16S rRNA sequencing on fecal samples from DSS-treated mice administered PM or 5-ASA. Beta diversity analysis (PCoA) revealed significant structural segregation between healthy controls and DSS-induced colitis mice (PERMANOVA, *p* < 0.05; Figure 6A). Notably, both PM and 5-ASA restored microbial community structure, clustering closely with healthy controls (Figure 6B). Alpha diversity metrics (Chao1, Shannon, Pielou’s evenness) confirmed reduced richness and diversity in colitis, which PM significantly rescued (Figure 6C and Figure S3A).

**Figure 7:**
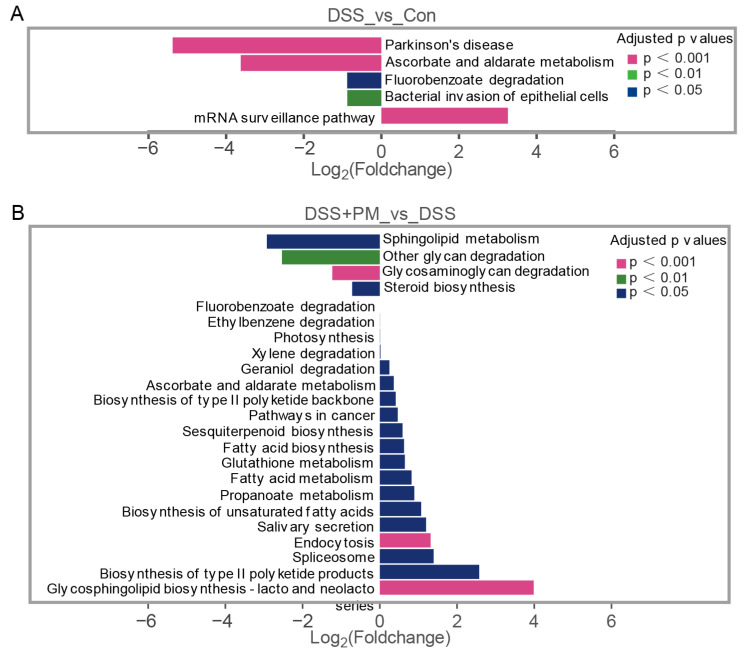
Enriched KEGG pathways from the composition of fecal bacteria according to functional genes. The differentially enriched pathways between (**A**) control and DSS group, and (**B**) between DSS and DSS + PM group.

Taxon-specific analysis demonstrated stark compositional shifts: DSS colitis enriched pathobionts (*Streptococcus*, *Escherichia*, *Alkaliphilus*), while depleting beneficial genera (*Akkermansia*, *Lactobacillus*, *Parabacteroides*; Figure 6D,E). PM administration reversed these changes, suppressing pathogens and restoring symbionts. Species-level profiling identified *Escherichia coli* and *Streptococcus equinus* as colitis-associated, whereas PM increased mucin-degrading *Akkermansia muciniphila* and short-chain fatty acid (SCFA)-producing *Lactobacillus* spp. (Figure S3B,C). LEfSe analysis further established *Enterobacteriaceae* (DSS group) versus *Muribaculaceae*/*Porphyromonadaceae* (PM/5-ASA groups) as key discriminators (Figure S4). Functional prediction (KEGG) linked colitis-associated microbiota to pathogenic pathways (bacterial epithelial invasion, LPS biosynthesis; Figure 7A and Figure S5A). Conversely, PM enriched barrier-protective and anti-inflammatory pathways, including sphingolipid metabolism, glycan degradation, and steroid biosynthesis (Figure 7B). Critically, PM suppressed pro-inflammatory sulfoglycolysis and aerobactin biosynthesis while enhancing propanoate fermentation (Figure S5B).

These findings indicate that DSS-induced epithelial damage facilitates pathogenic colonization, driving inflammation. PM counteracts this process by restoring microbiota homeostasis and redirecting microbial metabolism toward mucosal protection.

### 2.5. PM Restores DSS-Induced Impairment of Beneficial Short-Chain Fatty Acid Production

**Figure 8:**
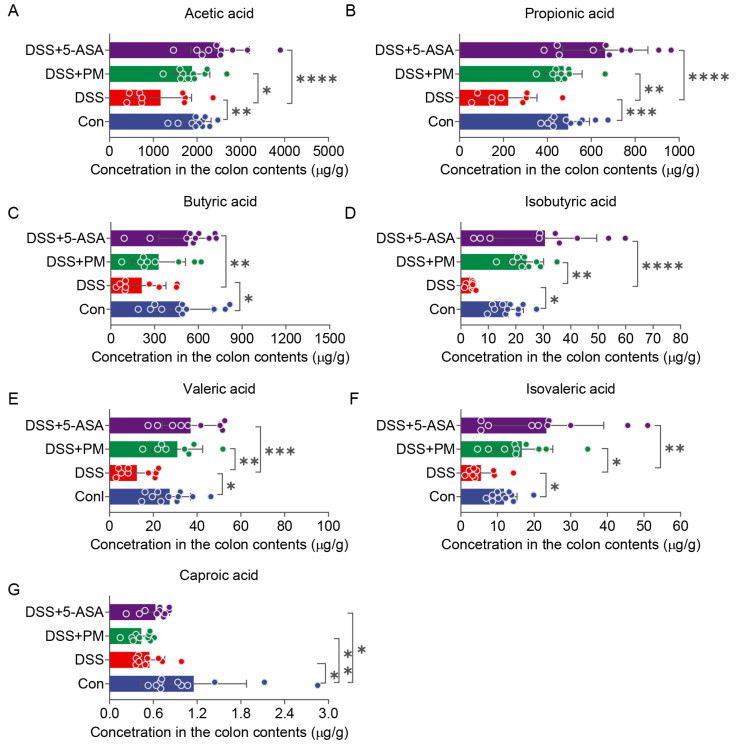
Concentrations of fecal (**A**) acetic acid, (**B**) propionic acid, (**C**) butyric acid, (**D**) isobutyric acid, (**E**) valeric acid, (**F**) isovaleric acid, and (**G**) caproic acid upon oral therapy. * *p* ≤ 0.05, ** *p* ≤ 0.01, *** *p* ≤ 0.001, and **** *p* ≤ 0.0001 indicate significant differences between different groups.

To investigate the impact of peimine (PM) on microbial metabolic output, we quantified fecal concentrations of seven short-chain fatty acids (SCFAs): acetic, propionic, butyric, isobutyric, valeric, isovaleric, and caproic acids. DSS administration significantly reduced levels of acetic acid and propionic acid compared to controls. Notably, both PM and 5-ASA treatment restored their production to near-normal levels (Figure 8A,B). Similarly, DSS-induced depletion of butyric, valeric, isobutyric, and isovaleric acids was significantly reversed by PM and 5-ASA supplementation (Figure 8C–F). In contrast, while caproic acid levels were also suppressed in the DSS group, neither PM nor 5-ASA restored its production (Figure 8G).

## 3. Discussion

Building upon our prior demonstration of aged garlic’s efficacy in murine colitis [16], this study identifies peimine (PM)—a bioactive compound significantly upregulated in aged garlic through non-targeted metabolomics—as a key therapeutic candidate. Network pharmacology analysis predicted PM’s interaction with ulcerative colitis (UC) targets primarily through modulation of PI3K-AKT and JAK-STAT signaling pathways (Figure S6), prompting further mechanistic investigation. The PI3K-AKT pathway, critically implicated in UC pathogenesis [20,21], appears modulated by PM through binding to PIK3R1 and AKT1. This interaction likely inhibits pathological hyperactivation of the pathway, thereby attenuating immune cell infiltration, preventing tissue damage, and restoring intestinal barrier integrity via enhanced mucosal repair [22]. Concurrently, PM’s potential regulation of JAK-STAT signaling—a canonical inflammatory mediator in UC—aligns with our experimental observations where PM significantly suppressed DSS-induced elevations in key pro-inflammatory cytokines (IL-6, TNF-α) in both serum and colonic tissue [22,23,24,25,26]. This cytokine reduction coincided with reduced neutrophil/B-cell infiltration and preserved epithelial architecture. While KEGG enrichment analysis strongly supports these pathways as primary targets, the predictions require orthogonal validation through methods such as Western blot or qPCR. Notwithstanding this limitation, the concordance between computational predictions and observed in vivo anti-inflammatory outcomes establishes a robust mechanistic framework for future studies.

Beyond its established anti-inflammatory properties, our findings reveal peimine (PM) as a potent modulator of oxidative stress in ulcerative colitis—a pathological process intimately linked to UC progression [27]. Network pharmacology predictions identified PM’s potential engagement with the HIF-1 signaling pathway, suggesting a mechanistic basis for its antioxidative actions. Critically, we demonstrate that PM administration significantly enhances colonic activities of key antioxidant enzymes: superoxide dismutase (SOD) and glutathione peroxidase (GSH-Px). This restoration is functionally significant; SOD deficiency exacerbates oxidative damage and colitis severity, while GSH-Px constitutes a vital mucosal antioxidant barrier [28,29]. Concordantly, PM suppressed colitis-associated biomarkers of oxidative injury—notably reducing myeloperoxidase (MPO) (reflecting neutrophil infiltration) and malondialdehyde (MDA) (indicating lipid peroxidation) [30,31]. This dual enhancement of endogenous antioxidants and attenuation of oxidative damage markers underscores PM’s capacity to counteract the redox imbalance central to UC pathogenesis.

Substance P (SP), abundantly expressed in the gut and integral to neuro-immune crosstalk, exacerbates colitis by activating NK-1R to drive inflammatory cell infiltration and cytokine release [32,33]. Our data align with this paradigm: DSS-induced colitis significantly elevated SP levels in serum and colon. Notably, PM administration selectively downregulated serum SP, while 5-ASA reduced SP in both compartments, suggesting distinct mechanisms of action. Vasoactive intestinal peptide (VIP), conversely, exerts vasodilatory and anti-inflammatory effects critical for epithelial barrier maintenance [34]. Exogenous VIP administration attenuates colitis by preserving tight junctions and restoring gut homeostasis [35,36,37]. Intriguingly, despite its protective role, both serum and colonic VIP levels surged in DSS mice—a compensatory response potentially overwhelmed by inflammation. Both PM and 5-ASA effectively normalized this dysregulation. Finally, 5-hydroxytryptamine (5-HT), primarily derived from enterochromaffin cells, potentiates inflammation via immune cell activation and proinflammatory cytokine secretion when dysregulated [38]. We observed significant 5-HT elevations in serum and colon during colitis. While PM selectively suppressed colonic 5-HT, neither intervention fully normalized serum levels, indicating compartment-specific modulation of this pathway. Collectively, our data suggest a potential association between PM treatment and the modulation of neuro-immune mediators, which might contribute to the observed anti-inflammatory effects, although a direct causal relationship remains to be established.

In vertebrates, commensal probiotics such as *Faecalibaculum*, *Bifidobacterium*, and *Parabacteroides* play pivotal roles in maintaining gut homeostasis through fermentation, pH regulation, SCFA production, epithelial crosstalk, and immunomodulation [39,40,41,42]. Our study revealed a marked dysbiosis in DSS-induced colitis, characterized by depletion of genera (*Akkermansia*, *Pediococcus*, *Faecalibaculum*, *Hungatella*, *Porphyromonas*, *Parabacteroides*, *Lactobacillus*, *Clostridium*, and *Bifidobacterium*) and concomitant enrichment of pathobionts (*Streptococcus* and *Escherichia*). The observed increase in the abundance of the genus *Clostridium*, coupled with the concurrent rise in fecal SCFA levels (particularly butyrate), suggests a potential shift towards a microbial community with enhanced capacity for beneficial metabolite production. However, as 16S rRNA sequencing does not resolve to the species level, this inference should be interpreted with caution. Critically, PM treatment reversed this dysbiotic signature, suppressing *Streptococcus* and *Escherichia* while significantly restoring *Akkermansia* and *Lactobacillus* populations. Functional analysis indicated that colitis-enriched bacteria contribute to mucosal barrier disruption and neurological complications [43], whereas PM-modulated taxa were strongly associated with glycolytic metabolism and epithelial protection—notably through sphingolipid metabolism, a key pathway in barrier integrity, but which requires direct experimental validation in future studies. This microbial remodeling directly correlated with SCFA dynamics: DSS-induced depletion of multiple SCFAs (acetate, propionate, butyrate, valerate, isobutyrate, isovalerate) was significantly rescued by PM treatment. Collectively, these findings demonstrate that PM promotes gut health by enriching SCFA-producing symbionts that reinforce mucosal barrier function and suppress inflammation.

Collectively, our study establishes that PM mitigates ulcerative colitis through a multi-mechanistic framework targeting intestinal inflammation, oxidative stress, and microbial dysbiosis. PM administration restores gut barrier integrity by enriching beneficial taxa (*Akkermansia muciniphila*, *Lactobacillus* sp. *L-YJ*) while suppressing pathogenic bacterial expansion. Concurrently, PM modulates core signaling pathways (PI3K-AKT, JAK-STAT, HIF-1) to attenuate inflammatory cascades and oxidative damage. Critically, these actions synergize to promote mucosal healing and sustain intestinal homeostasis, as evidenced by restored short-chain fatty acid production and histological recovery. Our findings position PM as a novel therapeutic agent with significant translational potential for UC management, offering a dietary-derived strategy to address the multifactorial pathogenesis of inflammatory bowel disease.

## 4. Materials and Methods

### 4.1. Network Pharmacological Analysis

The potential targets of PM were predicted by submitting its canonical SMILES string (C[C@H]1CC[C@H]2[C@@]([C@H]3CC[C@@H]4C@HC[C@H]5[C@H]4C[C@@H]([C@@H]6[C@@]5(CCC@@HO)C)O)(C)O) to the Swiss Target Prediction database (http://swisstargetprediction.ch/ (accessed on 11 November 2025)) and the SuperPred database (http://bioinformatics.charite.de/superpred (accessed on 17 November 2025)). For the SwissTargetPrediction results, all targets with a probability greater than 0 were selected; for the SuperPred results, all targets with a probability greater than or equal to 50% were selected (Supplementary Tables S1 and S2). The targets from both databases were then combined, and duplicates were removed to yield the final set of potential targets for PM. The Gene Cards database (https://www.genecards.org/ (accessed on 11 November 2025)) was searched for “ulcerative colitis”. Venny 2.1 software (https://bioinfogp.cnb.csic.es/tools/venny/ (accessed on 11 November 2025)) was used to determine the common targets of PM and UC.

The Gene Ontology (GO) and Kyoto Encyclopedia of Genes and Genomes (KEGG) of common targets were analyzed by the DAVID database (https://davidbioinformatics.nih.gov/ (accessed on 11 November 2025)). Visualization of enrichment analysis was performed using the Wei Sheng Xin (https://www.bioinformatics.com.cn/ (accessed on 11 November 2025)).

The STRING database (https://cn.string-db.org/ (accessed on 11 November 2025)) was used to construct the protein-protein interaction (PPI) network (species: “*Homo sapiens*”, confidence > 0.7) of the common targets [44]. The PPI network was visualized using Cytoscape 3.91 software, and the network’s nodes were evaluated for degree, betweenness centrality (BC), and closeness centrality (CC). The key targets were screened by first ranking the nodes in descending order of degree value, and then selecting the top ten targets that exhibited both BC and CC values above the average thresholds (BC > 0.2383, CC > 0.4673).

Molecular docking predicts how a receptor and ligand bind by analyzing their properties and interactions. The Protein Data Bank (PDB, https://www.rcsb.org/ (accessed on 11 November 2025)) was used to determine the protein’s 3D structure, and the PubChem database was used to identify the PM’s 3D structure. AutoDock 1.5 and AutoDock Vina 1.1 software were utilized to prepare the protein and ligands and conduct the docking calculations. Binding affinity was evaluated based on energy, with lower values indicating stronger binding. The predicted inhibition constant (pKi) was calculated from the binding free energy (ΔG) using the thermodynamic relationship: Ki = exp (ΔG/(R × T)), where R is the gas constant (1.987 cal/mol·K) and T is the absolute temperature (298 K). The pKi value, defined as −log_10_ (Ki), provides a quantitative measure of inhibitory potency, with higher pKi values indicating stronger inhibition [45]. The best docking results were selected and visualized using LigPlot+ 2.2 and PyMOL 3.1 software. Gene expressions of predicted targeted proteins were detected by quantitative RT-PCR in colon tissues in vivo experiment.

### 4.2. Mice and Experiment Design

48 seven-week-old specific pathogen-free (SPF) male C57BL/6 J mice weighing 18–23 g (SPF Biotechnology Co., Ltd., Beijing, China) were housed in a standard SPF facility with a 12 h light/dark cycle at 22 °C, and free access to food and water. Colitis was induced by administering 2.0% DSS (molecular mass 36–50 kDa, MP Biologicals, Solon, OH, USA) through drinking water. Following a 1-week acclimation period, mice were randomly allocated into four groups (n = 12 per group, 4 mice in one cage). According to our preliminary study (Figure S1), groups were treated as follows: [1] Oral-control (Con): sterile water for 7 days, PBS for 14 days at 8:00 AM daily; [2] DSS + Oral-PBS: 2.0% DSS for 7 days, PBS for 14 days at 8:00 AM daily; [3] DSS + Oral-PM: 2.0% DSS for 7 days, 4 mg/kg peimine (PM, P23700, purity ≥ 98%, Hangzhou Chuangtry Biological Technology Co., Ltd., Hangzhou, China) for 14 days at 8:00 AM daily; [4] DSS + Oral-5-ASA: 2.0% DSS for 7 days, 0.1 g/kg 5-acetylsalicylic acid (5-ASA, purity ≥ 98%, Hangzhou Chuangtry Biological Technology Co., Ltd., Hangzhou, China) for 14 days at 8:00 AM daily.

Body weight was measured daily, and disease activity index (DAI) assessed colitis severity based on weight loss, diarrhea, and blood in feces. On day 14, mice were sacrificed after general anesthesia with isoflurane (5%, for veterinary use) inhalation; colon length was measured, fecal samples were stored at −80 °C, and serum was collected by centrifugation (3000 rpm, 15 min, 4 °C) for future analysis. Colon tissue was fixed in 4% paraformaldehyde for histology and snap-frozen in liquid nitrogen for molecular analysis. Organ indices of the thymus, spleen, liver, kidney, and testis were calculated.

The study protocols were approved by the Ethical Committee of Animal Experiments, Hunan Agricultural University (approval no. 2021.050, approval date 1 May 2021).

### 4.3. Histological Analysis

After fixation in 4% paraformaldehyde for 24 h, colon samples were trimmed, dehydrated, embedded in paraffin, and sectioned into 5 μm slices. For histological observation, sections were dewaxed, rehydrated, and stained with hematoxylin (Zhongshan Golden Bridge Biotechnology Co., Ltd., Beijing, China) for 5 min. After rinsing with distilled water, sections were treated with 1% HCl in 75% alcohol for 20 s, incubated in PBS for 10 min, and then stained with eosin (Zhongshan Golden Bridge Biotechnology Co., Ltd., Beijing, China) for 30 s. After destaining in 95% alcohol for 1 min, sections were dehydrated in 100% alcohol, cleared in xylene, and mounted for microscopic examination. Mucosal height measurements were performed by a single investigator who was blinded to the experimental groups. All histological sections were assigned random codes prior to analysis to ensure objective assessment.

### 4.4. Periodic Acid-Schiff Stain

The paraffin sections were dewaxed, rehydrated, and stained with periodic acid fluid for 10 min. After rinsing with distilled water, the sections were stained with Schiff solution for 10 min, and washed with distilled water. Then the sections were dehydrated in 70%, 80%, 90%, 100% alcohol followed by xylene and were mounted by permount ^TM^ mounting medium for light microscopic observations.

### 4.5. Quantification of Inflammatory Cytokines and Antioxidant Indexes in the Colon and Serum

Frozen colon samples were homogenized with RIPA lysis buffer (Solarbio, Beijing, China) to extract total proteins, and then centrifuged at 1000 rpm at 4 °C for 15 min. Protein concentrations were measured using a BCA protein assay kit (Solarbio, Beijing, China). Serum and colon homogenate were analyzed for interleukin-6 (IL-6, ml063159), tumor necrosis factor-α (TNF-α, ml002095), IL-1β (ml098416), P substance (SP, ml001885), vasoactive intestinal peptide (VIP, ml001911), and 5-hydroxytryptamine (5-HT, ml001891) using ELISA kits (Shanghai Enzyme-linked Biotechnology Co., Ltd., Shanghai, China). Redox enzyme levels of myeloperoxidase (MPO), total superoxide dismutase (T-SOD), glutathione peroxidase (GSH-px), and malondialdehyde (MDA) were quantified using kits from Nanjing Jiancheng Bioengineering Institute (Nanjing, China).

### 4.6. Microbial Amplicon Sequencing and Data Analysis

Total microbial genomic DNA was extracted from colon contents using the OMEGA Stool DNA Kit (M4015-02, Omega Bio-Tek, Norcross, GA, USA), and DNA concentration and quality were assessed with a NanoDrop NC2000 spectrophotometer (ThermoFisher Scientific, Waltham, MA, USA) and agarose gel electrophoresis. PCR amplification of nearly full-length 16S rRNA genes was performed with primers 27F and 1492R, using Q5 reaction buffer, Q5 High-Fidelity DNA Polymerase, and dNTPs (New England Biolabs, MA, USA). PCR amplicons were purified with Agencourt AMPure Beads (Beckman Coulter, Indianapolis, USA) and quantified using the PicoGreen dsDNA Assay Kit (Invitrogen, Carlsbad, CA, USA). Sequencing was carried out on the PacBio Sequel platform (Shanghai Personal Biotechnology Co., Ltd., Shanghai, China) with Circular Consensus Sequencing (CCS) to minimize errors. Microbiome analysis was performed using QIIME2 (2022.11), with sequences processed using the DADA2 plugin for denoising, chimera removal, and alignment with MAFFT. Alpha- and beta-diversity metrics were calculated, and taxonomy was assigned using the classify-sklearn classifier against the NCBI/SILVA Release 138 Database. Differentially abundant taxa were identified with LEfSe, and microbial functions were predicted using PICRUSt2 and the MetaCyc and KEGG databases.

### 4.7. Short-Chain Fatty Acid (SCFA) Quantitative Analysis

Samples were homogenized for 1 min with 500 μL of water and 100 mg of glass beads, and then centrifuged at 4 °C for 10 min at 12,000 rpm. 200 μL of the supernatant was mixed with 100 μL of 15% phosphoric acid, 20 μL of 375 μg/mL 4-methylvaleric acid as an internal standard, and 280 μL ether. After vortexing for 1 min and centrifuging again at 4 °C for 10 min at 12,000 rpm, the supernatant was transferred into a vial for Gas Chromatography-Mass Spectrometry (GC-MS) analysis.

GC analysis was performed using a Trace 1300 gas chromatograph (Thermo Fisher Scientific, Waltham, MA, USA) with an Agilent HP-INNOWAX capillary column (30 m × 0.25 mm ID × 0.25 μm). Helium was used as the carrier gas at 1 mL/min. Injection was in split mode (10:1) with a 1 μL volume and an injector temperature of 250 °C. The ion source and MS transfer line were maintained at 300 °C and 250 °C, respectively. The column temperature was programmed from 90 °C to 120 °C at 10 °C/min, then to 150 °C at 5 °C/min, and finally to 250 °C at 25 °C/min, held for 2 min.

Mass spectrometric detection was performed on an ISQ 7000 (Thermo Fisher Scientific, Waltham, MA, USA) in electron impact ionization mode (70 eV) using Single Ion Monitoring (SIM). The content of acetic acid, propionic acid, isobutyric acid, butyric acid, isovaleric acid, and valeric acid was calculated using the formula:

(1) $$\mathrm{Content}\;\left(\mu g/g\right)\;=\;\frac{C\;\left(\mu g/\mathrm{mL}\right)\;\times \;0.5}{\mathrm{Amount}\;\left(\mathrm{mg}\right)}\;\times \;1000$$

For caproic acid:

(2) $$\mathrm{Content}\;\left(\mu g/g\right)\;=\;\frac{C\;\left(\mu g/\mathrm{mL}\right)\;\times \;0.5\;\times \;1.5}{\mathrm{Amount}\;\left(\mathrm{mg}\right)}\;\times \;1000$$

Standard solutions ranged from 0.02 to 500 μg/mL.

### 4.8. Statistical Analysis

All statistical analyses were performed using Prism 9.0 (GraphPad Software). Data are expressed as means ± standard error (SD). Statistical significance was evaluated using one-way ANOVA for Tukey’s multiple comparisons test, and Student’s t-test for paired comparisons. Asterisk coding is indicated as * *p* ≤ 0.05, ** *p* ≤ 0.01, *** *p* ≤ 0.001, and **** *p* ≤ 0.0001.

## Figures and Tables

**Table 1:** Degree, closeness, betweenness and binding energy of key targets for peimine.

Name	Degree ^1^	Closeness Centrality ^2^	Betweenness Centrality ^3^	Binding Energy (kcal/mol)	Predicted Inhibitory Constant (pKi)
STAT3	28	0.68	0.21	−8.1 ± 0.0	5.94 ± 0.00 μM
HSP90AA1	24	0.63	0.10	−7.7 ± 0.2	5.65 ± 0.13 μM
AKT1	23	0.63	0.06	−9.4 ± 0.0	6.89 ± 0.00 μM
JAK2	23	0.63	0.08	−8.2 ± 0.0	6.01 ± 0.00 μM
ESR1	19	0.58	0.06	−8.1 ± 0.0	5.94 ± 0.00 μM
NFKB1	17	0.57	0.06	−7.5 ± 0.0	5.50 ± 0.00 μM
CXCR4	17	0.57	0.15	−7.2 ± 0.1	5.31 ± 0.08 μM
PIK3R1	17	0.55	0.04	−9.6 ± 0.0	7.04 ± 0.00 μM
TLR4	16	0.56	0.08	−7.5 ± 0.3	5.53 ± 0.18 μM
HIF1A	16	0.55	0.02	−7.6 ± 0.4	5.55 ± 0.30 μM

^1^ Degree: quantifies the direct connections of a node. ^2^ Closeness centrality: representing how quickly a node can communicate with others. ^3^ Betweenness centrality: measures the extent to which a node acts as a bridge or critical intermediary along shortest paths between nodes.

## Data Availability

The original contributions presented in this study are included in the article/Supplementary Materials. Further inquiries can be directed to the corresponding authors.

## Supplementary Materials

The following supporting information can be downloaded at: https://www.mdpi.com/article/10.3390/ijms262211203/s1.
