# Supplementary material for: Peimine Alleviates DSS-Induced Colitis by Modulating Gut Microbiota and Attenuating Inflammation and Oxidative Stress
Source: Int J Mol Sci. 2025 Nov 20;26(22):11203. doi: 10.3390/ijms262211203 (PMC12653015; doi:10.3390/ijms262211203)
Supplement: Supplementary file 1 [file ijms-26-11203-s001.zip › Supplementary Figures.pdf]

Supplemental information

Peimine Alleviates DSS-induced Colitis by Modulating Gut Microbiota and Attenuating Inflammation and Oxidative Stress

Xuke Guan, Deping Han, Haojie Sha Moyue Yao, Jiaying Zhang, Guangyao Zhang, Yibing Wu, Dingding Su and Qing Yang

Supplementary Figures S1-S6.

Supplementary figures

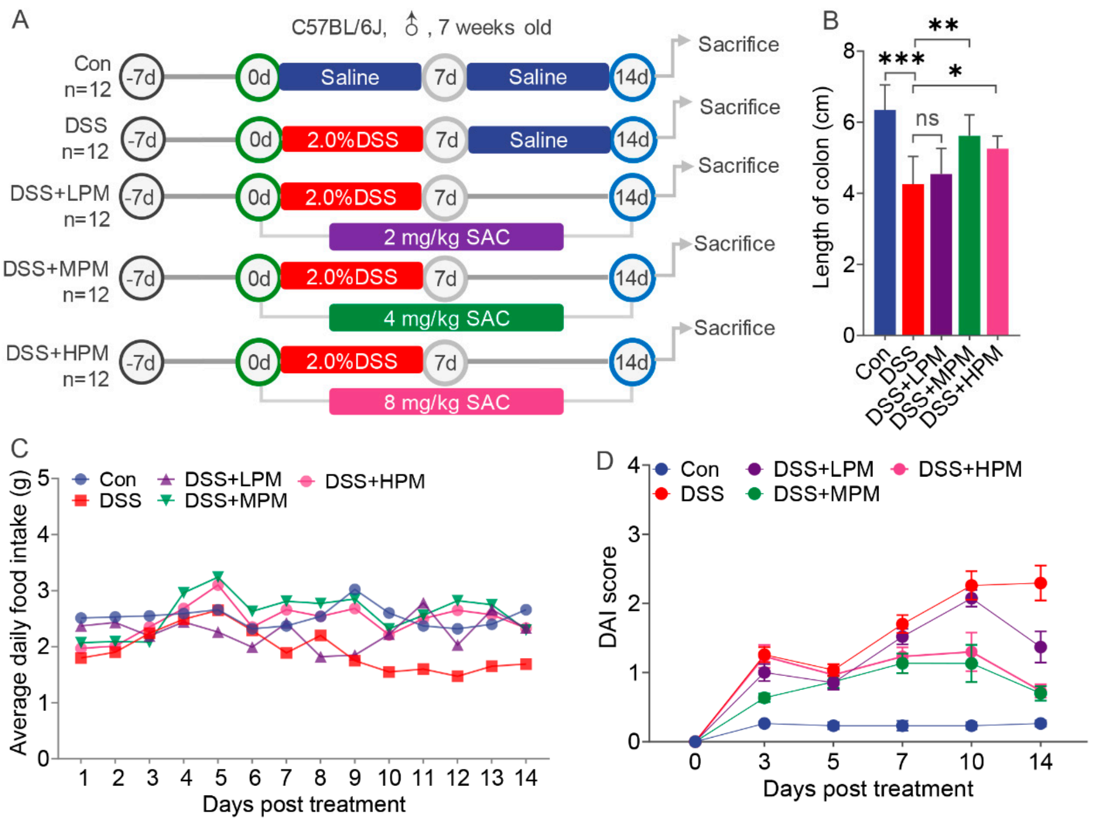

Figure S1. Oral different dose of PM alleviated DSS-induced experimental colitis. (A) Diagram illustrating the mouse model of colitis employed. (B) Length of colon from each group after treatment. (C) Daily food intake. (D) Disease activity index (DAI) score. $^{*}p \leq 0.05$, $^{**}p \leq 0.01$, and $^{***}p \leq 0.001$ indicate significant differences between different groups. PM: peimine.

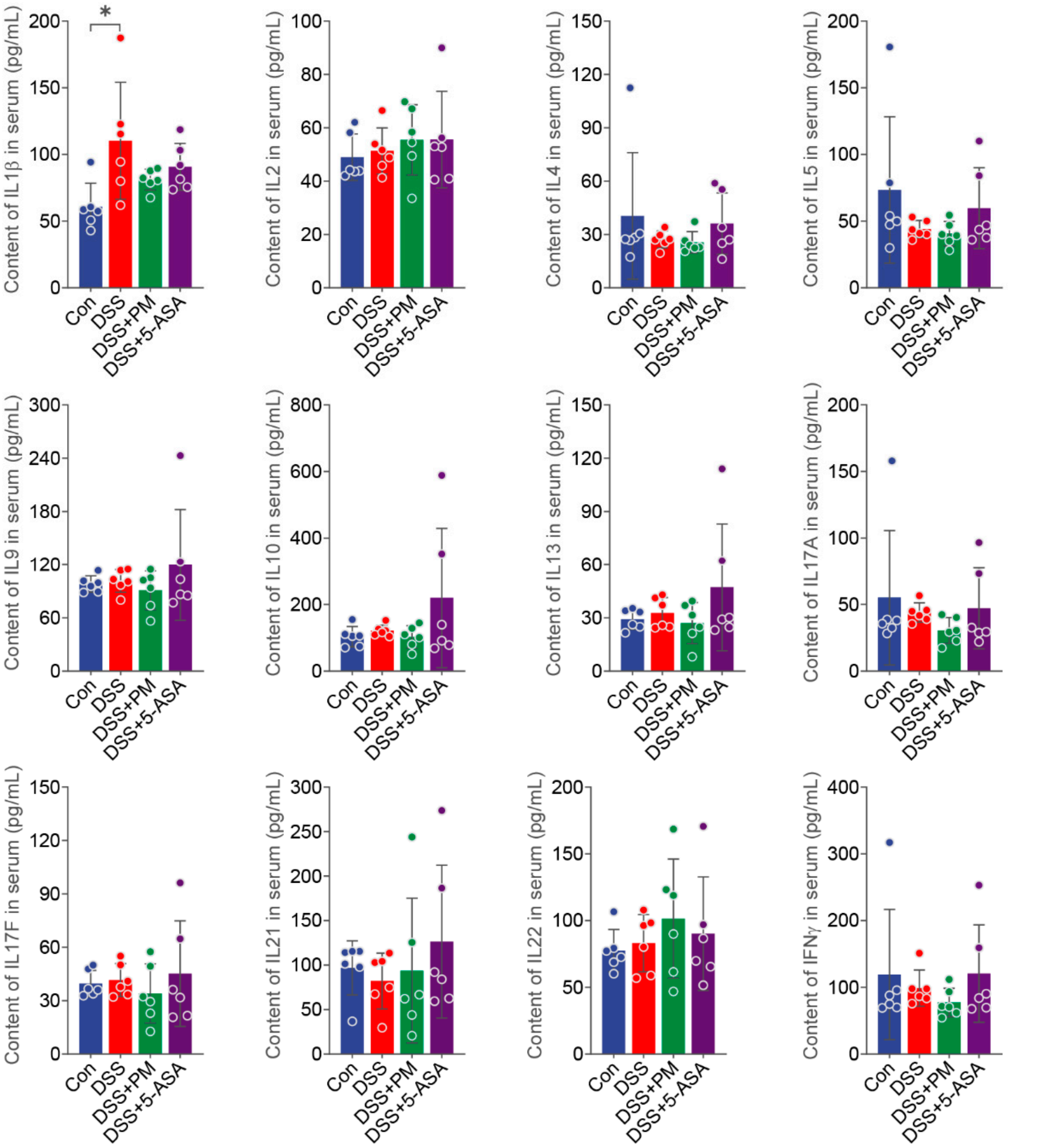

Figure S2. Comparison of cytokines in the serum between different treatments. $^{*}p \leq 0.05$ indicates significant differences between different groups.

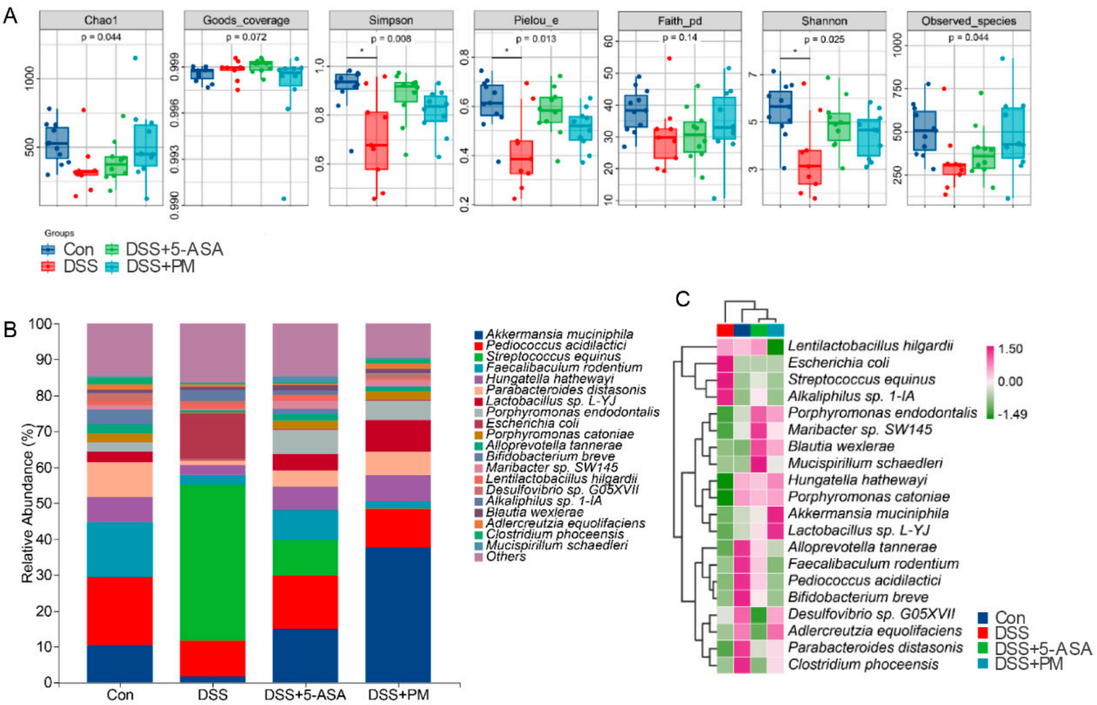

Figure S3. The relative abundance of fecal bacterium among different groups. (A) Alpha diversity in richness, diversity and evenness of fecal bacterium. (B) The relative abundance of top 20 bacterial species in each group. (C) Heatmap showed the composition and relative abundance of top 20 fecal bacteria species among groups.

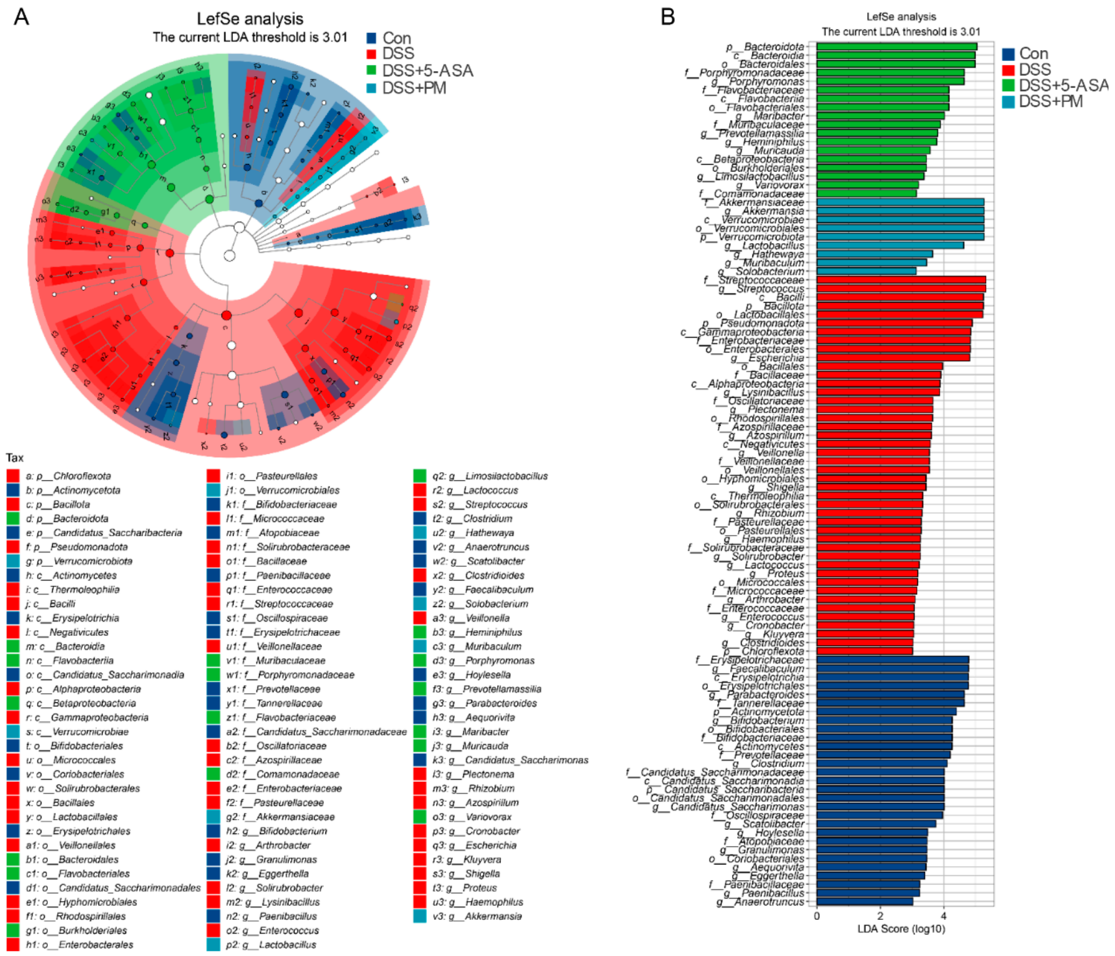

Figure S4. Lefse analysis of composition and relative abundance of fecal bacteria. (A) Cladogram showed the composition of bacteria induced by DSS, and attenuation of fecal bacteria after oral treatment of PM and 5-ASA. (B) Effect size rank of the relative abundance of differential bacteria species in each group.

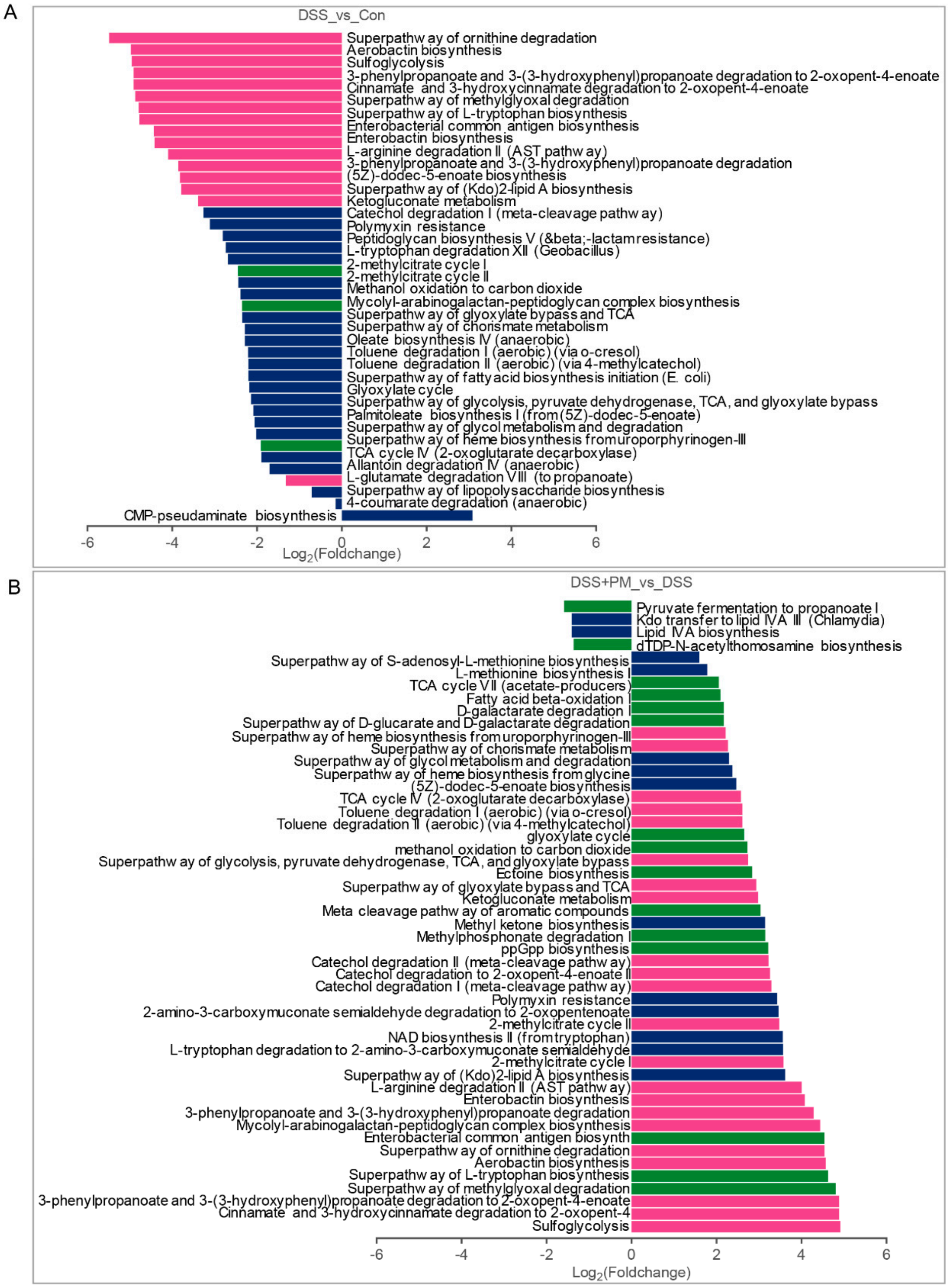

Figure S5. Enriched metabolic pathways from the composition of fecal bacteria according to functional genes. The differentially enriched pathways between control and DSS group (A), between DSS and DSS+PM group (B).

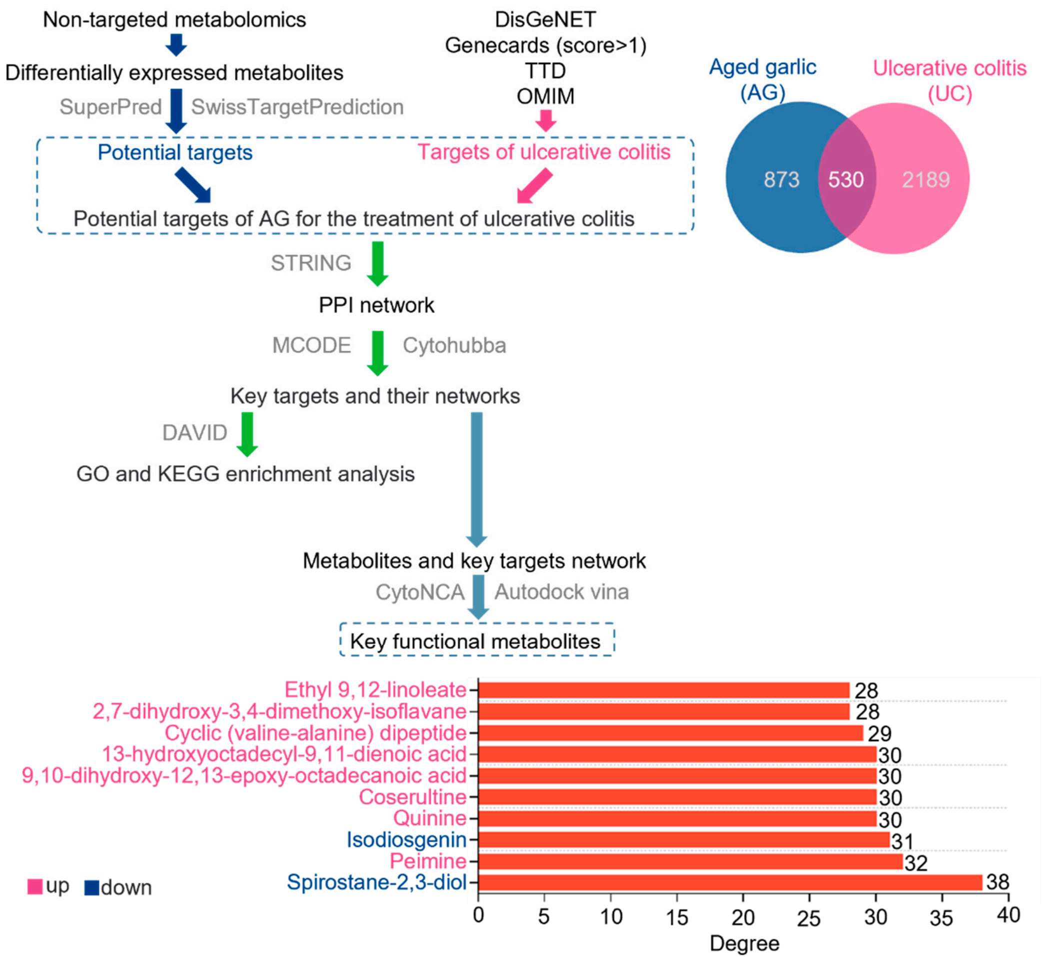

Figure S6. Network pharmacology analysis of target bioactive constituents in the AG on UC. AG: aged garlic; UC: ulcerative colitis.
